# Supplementary material for: CD103+ cDC1 Dendritic Cell Vaccine Therapy for Osteosarcoma Lung Metastases
Source: Cancers (Basel). 2024 Sep 24;16(19):3251. doi: 10.3390/cancers16193251 (PMC11482638; doi:10.3390/cancers16193251)
Supplement: Supplementary file 1 [file cancers-16-03251-s001.zip › cancers-3165579-supplementary.pdf]

Supplementary Data

CD103+ cDC1 dendritic cell vaccine therapy for osteosarcoma

Yuanzheng Yang[1], Yifan Zhou[2,3], Jian Wang[4], You Zhou[1], Stephanie S. Watowich[2] and

Eugenie S. Kleinerman*[,1, 5]

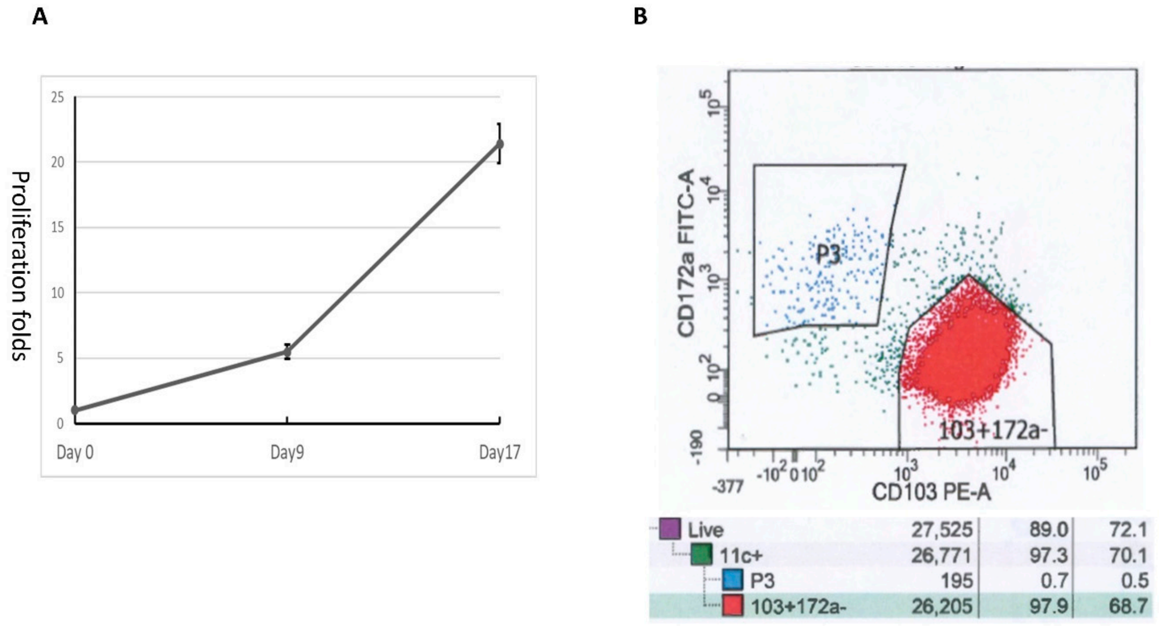

Figure S1. Ex vivo generation and expansion of CD103+ cDC1s cells from Balb/c mice. (A) Bone Marrow cells were expanded around 5 fold on day 9. Cell differentiated into DC cells by more than 20 fold on day 17. **(B)** FACS analysis of ex vivo cultured CD103+ cDC1 cells at day 17.

Supplemental Figure 2

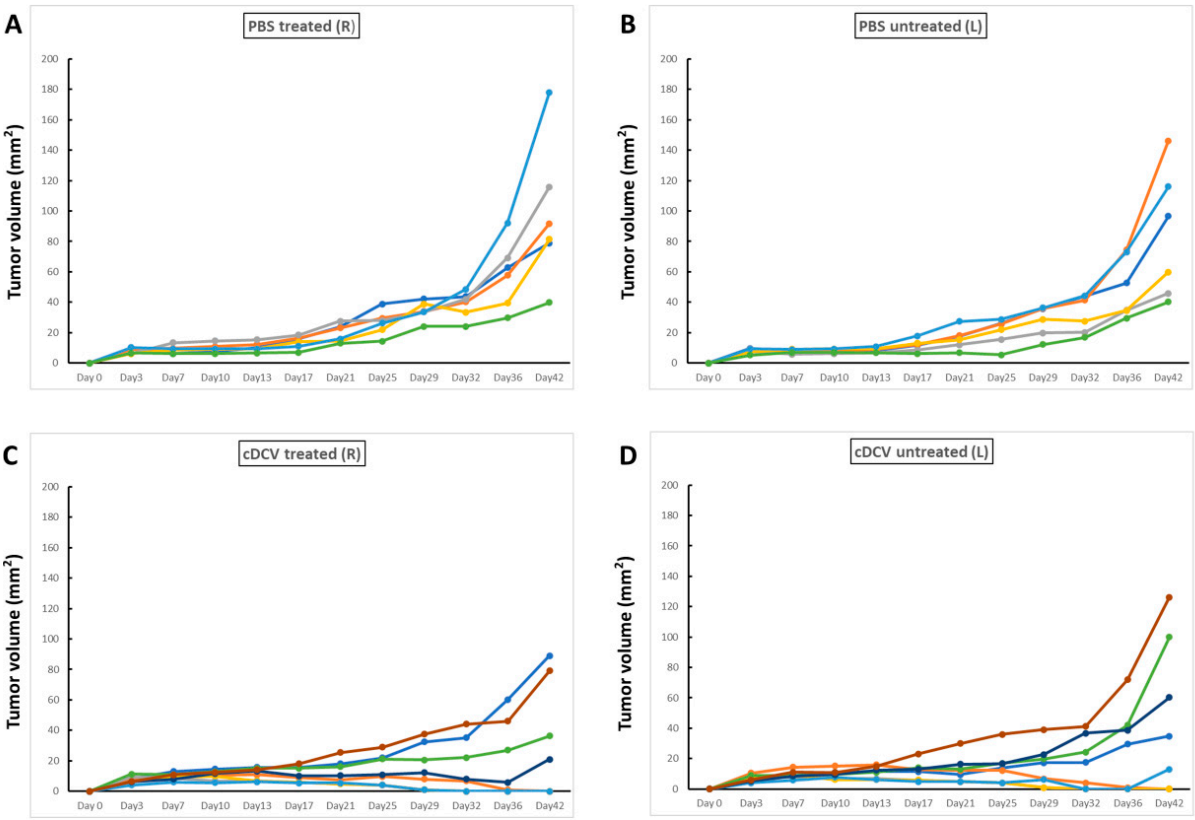

Figure S2. Effect of an intratumor cDCV generated from K7M3-tumor cell lysates on tumor growth and systemic immunity. $2{\times}10^6$ K7M3 cells were injected subcutaneously into the right and left sides of mice. On days 4 and 7, the right-side tumors were intratumorally injected with phosphate-buffered saline (PBS, control) or the type 1 CD103+ dendritic cell vaccine (cDCV). The left-side tumors were untreated. Tumor growth in the individual mice were assessed every 3-4 days for 5 weeks after therapy. **(A)** PBS treated (R), **(B**) PBS untreated (L), **(C)** cDCV treated (R) and **(D)** cDCV untreated (L).

A

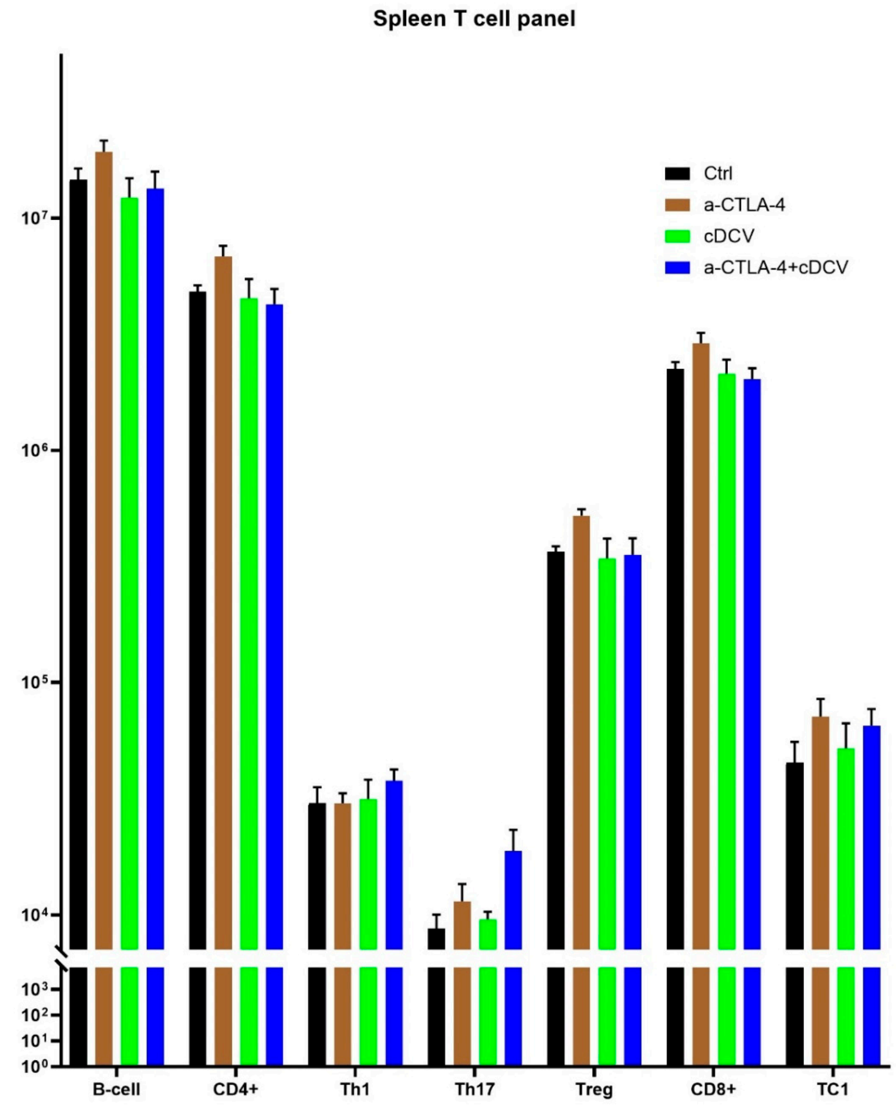

Spleen T cell panel
Ctrl
a-CTLA-4
cDCV
a-CTLA-4+cDCV
10^7
10^6
10^5
10^4
10^3
10^2
10^1
10^0
B-cell
CD4+
Th1
Th17
Treg
CD8+
TC1

B

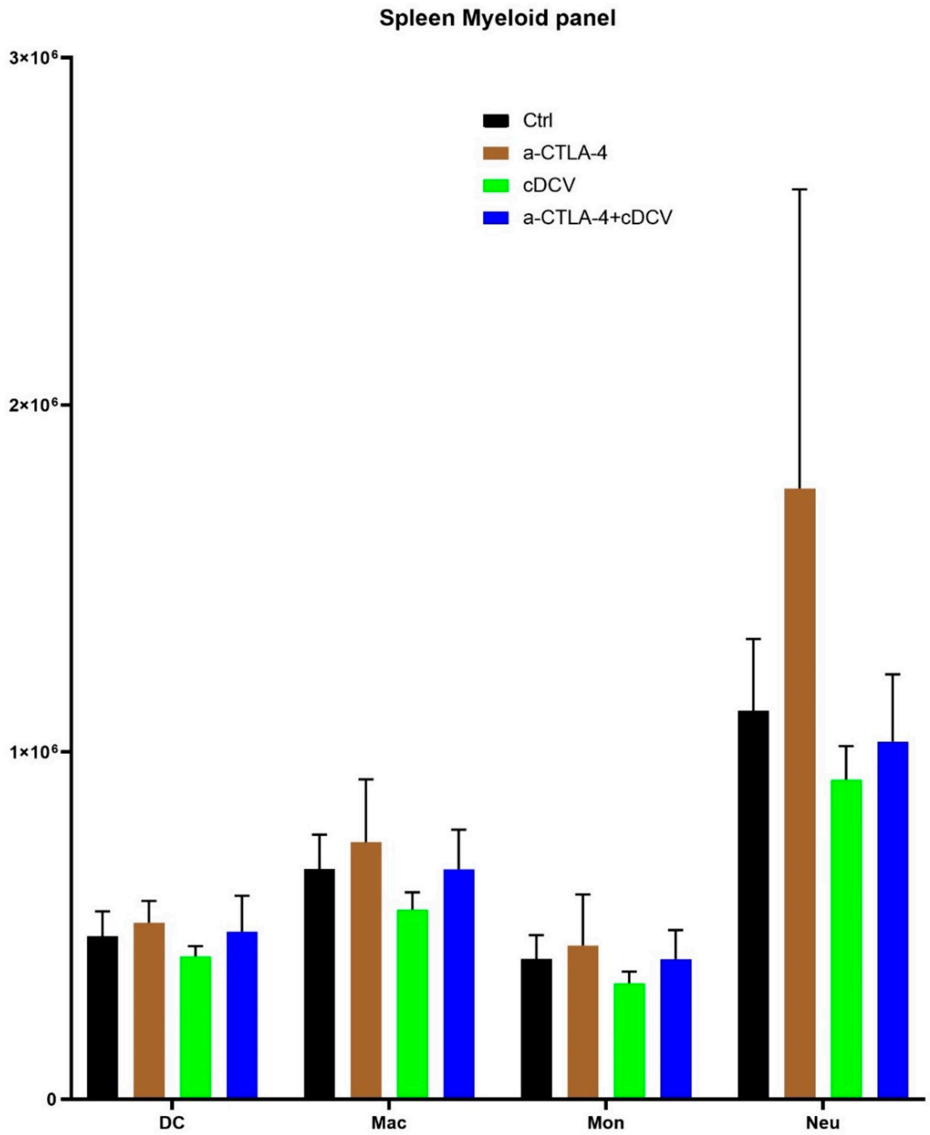

Figure S3. Effect of cDCV therapy with or without anti-CTLA-4 on immune cell profile in the spleen. Spleens from mice in Figure 4 were analyzed for **(A)** B-cell and T cell subsets, and **(B)** myeloid cells using fluorescence-activated cell sorting (FACS). cDCV, type 1 CD103$^+$ dendritic cell vaccine; CTLA-4, cytotoxic T lymphocyte antigen-4; Ctrl, control; DCs, dendritic cells; Macs, macrophages; Mons, monocytes; Neus, neutrophils; TC1s, cytotoxic T cells; Th1s, T-helper 1 cells; Th17s, T-helper 17 cells; Tregs, regulatory T cells.
